# Relationships Between Fatigue, Aggressiveness, Insomnia, and Sleep Quality Among Nurses

**DOI:** 10.1192/j.eurpsy.2025.2330

**Published:** 2025-08-26

**Authors:** A. M. Cybulska, K. Rachubińska, E. Grochans, I. Malicka-Szymoniak, D. Schneider-Matyka

**Affiliations:** 1 Department of Nursing, Pomeranian Medical University in Szczecin; 2 Independent Public Provincial Hospital in Szczecin, Szczecin, Poland

## Abstract

**Introduction:**

Nurses often face situations where they must deal with aggression from patients or even coworkers. This challenge can have negative consequences for both medical staff and patients.

**Objectives:**

The aim of this study is to examine the relationship between the level of aggression, the severity of fatigue, and the occurrence of sleep disorders among nursing staff.

**Methods:**

The study was conducted in Szczecin and was survey-based, involving 241 nurses working in surgical wards, medical wards, outpatient clinics, and the emergency department. The following tools were used to collect data: a custom survey, the Fatigue Severity Scale (FSS), the Athens Insomnia Scale (AIS), the Buss-Perry Aggression Questionnaire (BPAQ), and the Pittsburgh Sleep Quality Index (PSQI).

**Results:**

It was found that a greater tendency toward overall aggression, verbal aggression, and higher levels of anger and hostility were associated with more severe insomnia problems (p<0.05). Data analysis showed statistically significant correlations (p<0.05) between overall, physical, and verbal aggression, as well as hostility (based on BPAQ) and sleep quality (based on PSQI). A statistically significant positive correlation (p<0.05) was also found between fatigue levels (FSS) and sleep quality (PSQI) — the higher the level of fatigue, the worse the sleep quality.

**
Table 1. Comparison of results according to BPAQ and AIS, PSQI**

**Table:** 

Examined Traits	AIS		PSQI	
	r	p	r	p
BPAQ: Total Aggression	0,297	<0,001	0,227	<0,001
BPAQ: Verbal Aggression	0,143	0,026	0,147	0,022
BPAQ: Anger	0,202	0,002	0,118	0,068
BPAQ: Hostility	0,353	0,001	0,317	<0,001

**Conclusions:**

There is a link between aggression levels, fatigue, and sleep disorders. Individuals with stronger tendencies toward aggression were more likely to experience insomnia and sleep problems. Those with higher levels of fatigue also experienced more frequent insomnia and sleep disturbances. Preventive and therapeutic measures are necessary to improve the health of nursing staff.

**Disclosure of Interest:**

None Declared

